# The molecular phylogeny of *Chionaster nivalis* reveals a novel order of psychrophilic and globally distributed Tremellomycetes (Fungi, Basidiomycota)

**DOI:** 10.1371/journal.pone.0247594

**Published:** 2021-03-24

**Authors:** Nicholas A. T. Irwin, Chantelle S. Twynstra, Varsha Mathur, Patrick J. Keeling

**Affiliations:** 1 Department of Botany, University of British Columbia, Vancouver, British Columbia, Canada; 2 Merton College, University of Oxford, Oxford, United Kingdom; Leibniz Institut - Deutsche Sammlung von Mikroorganismen und Zellkulturen GmbH, GERMANY

## Abstract

Snow and ice present challenging substrates for cellular growth, yet microbial snow communities not only exist, but are diverse and ecologically impactful. These communities are dominated by green algae, but additional organisms, such as fungi, are also abundant and may be important for nutrient cycling, syntrophic interactions, and community structure in general. However, little is known about these non-algal community members, including their taxonomic affiliations. An example of this is *Chionaster nivalis*, a unicellular fungus that is morphologically enigmatic and frequently observed in snow communities globally. Despite being described over one hundred years ago, the phylogeny and higher-level taxonomic classifications of *C*. *nivalis* remain unknown. Here, we isolated and sequenced the internal transcribed spacer (ITS) and the D1-D2 region of the large subunit ribosomal RNA gene of *C*. *nivalis*, providing a molecular barcode for future studies. Phylogenetic analyses using the ITS and D1-D2 region revealed that *C*. *nivalis* is part of a novel lineage in the class Tremellomycetes (Basidiomycota, Agaricomycotina) for which a new order Chionasterales ord. nov. (MB838717) and family Chionasteraceae fam. nov. (MB838718) are proposed. Comparisons between *C*. *nivalis* and sequences generated from environmental surveys revealed that the Chionasterales are globally distributed and probably psychrophilic, as they appear to be limited to the high alpine and arctic regions. These results highlight the unexplored diversity that exists within these extreme habitats and emphasize the utility of single-cell approaches in characterizing these complex algal-dominated communities.

## Introduction

Snow and ice in the arctic and high-alpine present an inhospitable environment that is typically non-permissive to eukaryotic life. However, during the melt season, elevated temperatures, melting, and the formation of liquid water can generate suitable habitats in which algae, fungi, and a variety of other microbial eukaryotes can thrive [1, 2]. Although transient, these communities are both biodiverse and environmentally impactful as they are important for nutrient cycling, primary production, and can accelerate glacial melting by decreasing surface albedo in a positive feedback loop [2–6]. Additionally, climate change and ice cap recession have threatened these communities, making it pertinent to characterize their biodiversity and community function [1, 7].

The primary constituents of snow communities are green algae from the order Chlamydomonadales, such as *Chlamydomonas*, *Chloromonas*, and *Chlainomonas*, whose photosynthetic pigments, particularly chlorophyll and red carotenoids such as astaxanthin, create coloured snow patches [1, 8]. These, along with other algae, are primary producers and provide a carbon source to diverse heterotrophs and saprotrophs, which typically include ciliates, cercozoans, meiofaunal metazoans, and an abundance of fungi [9–11]. Although algae predominate in these communities, other community members can be comparably abundant in certain microenvironments, such as cryoconite holes [12], where they can act as algal parasites and predators [9, 13], and may be important during the initial stages of community establishment [14]. Despite the importance of these additional members, previous research has focused largely on understanding algal population dynamics, species distributions, and their correlations with environmental and metabolic variables [5, 8, 12], leaving our understanding of the other eukaryotes within these communities limited.

Besides algae, fungi represent the most commonly documented and investigated component of snow communities. Molecular analyses using environmental DNA and high-throughput sequencing approaches have revealed that basidiomycetous yeast and chytrids dominate the fungal constituent of these communities, where they likely function as saprotrophs and parasites [5, 11, 15–17]. Fungal community composition is in part dependent on the presence of algae, but the relative abundances of fungal representatives may also depend on community maturity, location, and seasonal shifts (e.g., chytrids may be more prevalent during late spring when run-off volumes are high) [15, 16, 18]. Although there are multiple hypotheses regarding the ecological function of chytrids in these communities, less has been discussed about the role of basidiomycetes despite their higher relative abundance in many analyses (however, this can be biased by rDNA copy number and heterogeneity) [5, 9, 11, 15, 19]. Of those species identified using molecular techniques, the majority of basidiomycetous yeast tend to relate to genera classified as *Rhodotorula* (Pucciniomycotina) or *Cryptococcus* (Agaricomycotina) [15]. However, these genera are polyphyletic making these classifications ambiguous [20]. Moreover, a number of additional fungal genera are frequently observed by microscopy in snow communities, such as *Chionaster* and *Selenotila*, yet these genera lack higher order taxonomic classifications and molecular barcodes, so it is unclear how they fit into environmental DNA surveys [10, 21, 22]. One of the challenges of studying these fungi is that they are difficult to culture, perhaps owing to the unique habitats in which they reside, but they are also morphologically incomparable, and the efficacy of molecular techniques in characterizing their diversity remains unclear. Nonetheless, having accurate taxonomic identifications of fungal community members and linking microscopic and molecular studies will be key in deciphering community function and the ecological impact of these snow-associated fungi.

A clear example of a poorly characterized, yet potentially ecologically relevant snow-associated fungal species, is *Chionaster nivalis*. *Chionaster nivalis* is a morphologically conspicuous unicellular fungi, characterized by the presence of an often-central condensed cytoplasm and three to five radiating arms that give the species a star-like appearance [23, 24]. This distinctive morphology has permitted *C*. *nivalis* to be confidently observed globally by microscopic analysis in algal-based snow communities across Europe, Australia, North America, and Asia [9, 21, 24–28]. Within the snow pack, the distribution of *C*. *nivalis* correlates with the presence of algae and cyanobacteria, suggesting it may depend nutritionally on algal extrudates [29]. However, physical associations between *C*. *nivalis* and algal species have also occasionally been observed, suggesting a more active interaction may exist [9, 30, 31]. Additionally, despite its morphology, the higher order taxonomy of *C*. *nivalis* remains unknown and a source of confusion. Indeed, *C*. *nivalis* was originally described as a *Tetraidron* green alga by Bohlin (1893) before being re-classified as a fungus (*Incertae sedis*) by Wille (1904), which was later supported by additional observations made by Kol (1935) [21, 23, 24, 32]. Current opinions suggest that *C*. *nivalis* may be related to aquatic hyphomycetes which share a similar star-like appearance [25, 26], but hyphomycetes themselves are polyphyletic in molecular phylogenetic studies, indicating that the morphology can be convergent, and phylogenetic information and life cycle descriptions for *C*. *nivalis* remain unavailable for comparison [33–35].

Here we sought to investigate the phylogeny of *C*. *nivalis* in order to provide a better understanding of one of most recognizable yet poorly understood members of microbial snow communities world-wide. To this end, we isolated cells of *C*. *nivalis* from algae-dominated red-snow and sequenced the internal transcribed spacer (ITS) and D1-D2 region of the ribosomal RNA operon for phylogenetic analysis. Molecular phylogenies revealed that *C*. *nivalis* is not closely related to sampled aquatic hyphomycetes, but rather belongs to a newly characterized order (Chionasterales ord. nov. MB838717, Chionasteraceae fam. nov. MB838718) within the Tremellomycetes (Basidiomycota) which is sister to the Cystofilobasidiales and contains multiple environmental sequences. Analysis of these environmental sequences confirmed the global distribution of *C*. *nivalis* and the Chionasterales, and reaffirmed their psychrophilic nature, as closely related sequences were only observed from arctic and high-alpine samples, although the distribution of the Chionasterales extends from snow to soil and plant-dominated environments.

## Results and discussion

### Morphology and identification of *Chionaster nivalis*

**Fig 1:**
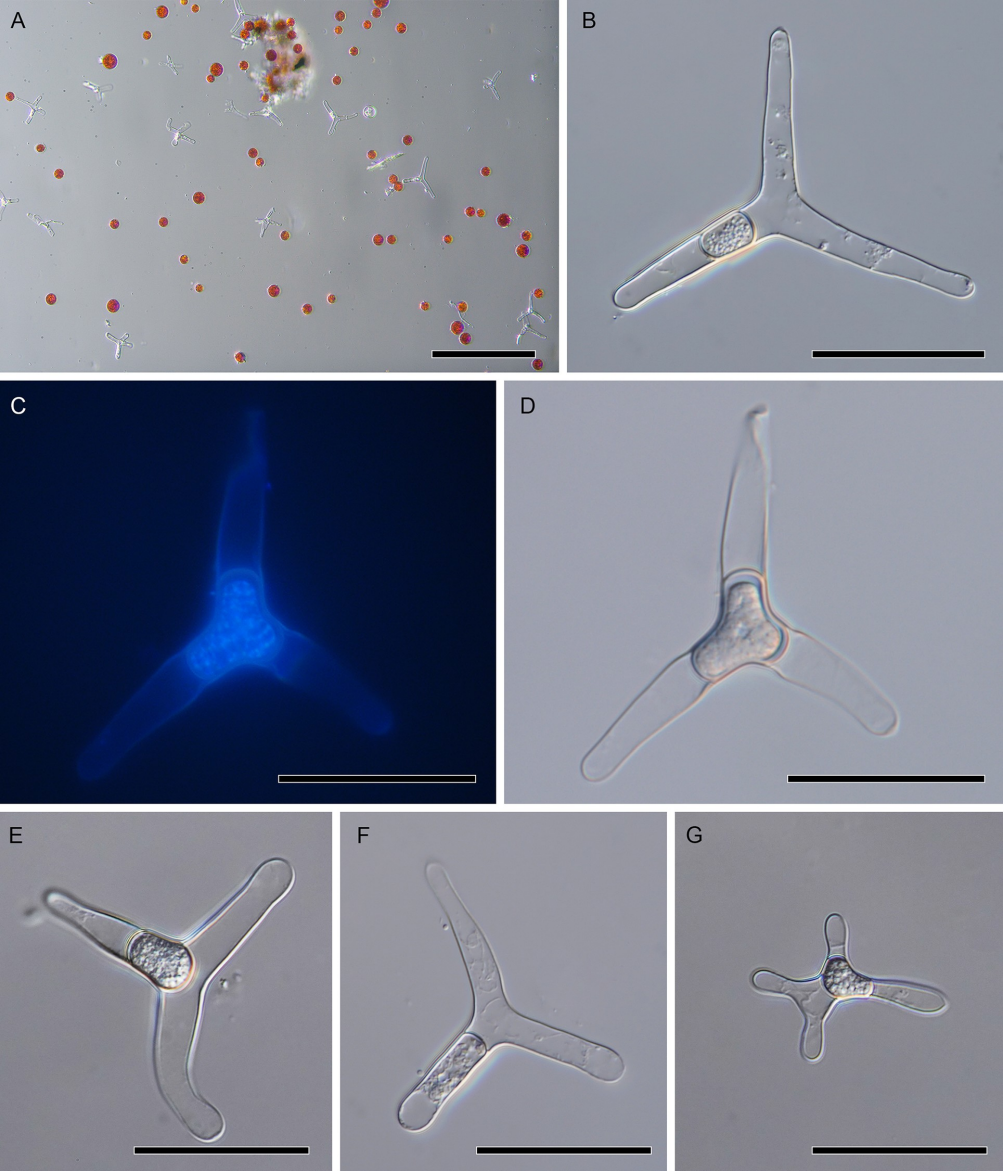
The morphology of *Chionaster nivalis*. **A.** An example of a red snow community with an above-average abundance of *C*. *nivalis*. Red algal cells likely represent *Chlamydomonas nivalis*. **B.** A light micrograph depicting the typical morphology of *C*. *nivalis*. Note the three radiating arms and globular cytoplasm. **C-D.** Fluorescent (**C**) and light (**D**) micrographs of a DAPI-stained individual. **E-G.** Light micrographs of *C*. *nivalis* demonstrating morphological variations such as bent extensions (**E**), a non-central cytoplasm (**F**), and a smaller four-armed individual (**G**). Scale bars: 150 μm (**A**), 30 μm (**B-G**).

Microscopic examination of red snow collected adjacent to Wedgemount Lake and Joffre Lake in the Coast Mountains of British Columbia, Canada, revealed the presence of *Chionaster nivalis* in all samples collected (Fig 1A). Identification of *C*. *nivalis* was made based on morphological comparisons to fungal species that have been characterized from snow communities in the Pacific Northwest and globally [21, 24]. Previous reports highlighted the presence of three species of fungi commonly observed in red snow in addition to chytrids, including *Chionaster nivalis*, *Chionaster bicornis*, and *Selenotila nivalis* [21]. The species from our samples were characterized by the presence of typically three to four thick radiating extensions with rounded ends and a cytoplasm which was often central or located in a single arm (Fig 1). The length of the extensions was typically around 30 μm, although smaller individuals were observed (Fig 1G). These observations align with previous accounts of *C*. *nivalis* and contrast with descriptions of *C*. *bicornis*, which has two long pointed horns which extend from a central cell, and *S*. *nivalis*, which is relatively small and usually has two to four spindle-shaped arms with attenuated ends [21, 24–26]. Therefore, the distinctive morphology of *C*. *nivalis* permits its confident identification.

Despite its presence, the abundance of *C*. *nivalis* was highly variable, even amongst adjacent sites, and the cells were often observed interspersed with algae (Fig 1A). Regardless of abundance and locality, *C*. *nivalis* morphology was consistent, although we observed variations in the appearance of the cytoplasm which was either homogeneous (Fig 1D–1F) or globular (Fig 1B and 1G). DNA staining and fluorescent imaging of *C*. *nivalis* with a homogenous cytoplasm revealed that the central body contains DNA and may represent the nucleus. However, staining was unsuccessful in individuals with alternative morphologies, reflecting either biological differences or technical limitations. Earlier reports have suggested that the globules observed in some individuals could reflect aplanospores or zoospores, the latter of which was used to suggest a relationship between *C*. *nivalis* and the Chytridiomycota [24, 31]. Indeed, we observed movement of these globules within the cell, though this may have reflected Brownian motion, particularly given their small size. More detailed and comprehensive observations of the lifecycle and morphology of *C*. *nivalis* will be required to properly characterize these cellular components.

### Phylogeny and biogeography of *Chionaster nivalis*

Although useful for species identification, the morphology of *C*. *nivalis* provides little taxonomic information. To reconcile this and provide a molecular barcode for the future identification of *C*. *nivalis* in environmental DNA surveys, we sequenced the internal transcribed spacer (ITS) of the ribosomal RNA (rRNA) operon and the D1-D2 region of the large subunit (LSU) rRNA gene, which are commonly used for fungal species classification and phylogeny [36, 37], from isolates (i.e., pools of cells) extracted from red snow collected near Wedgemount Lake, British Columbia.

**Fig 2:**
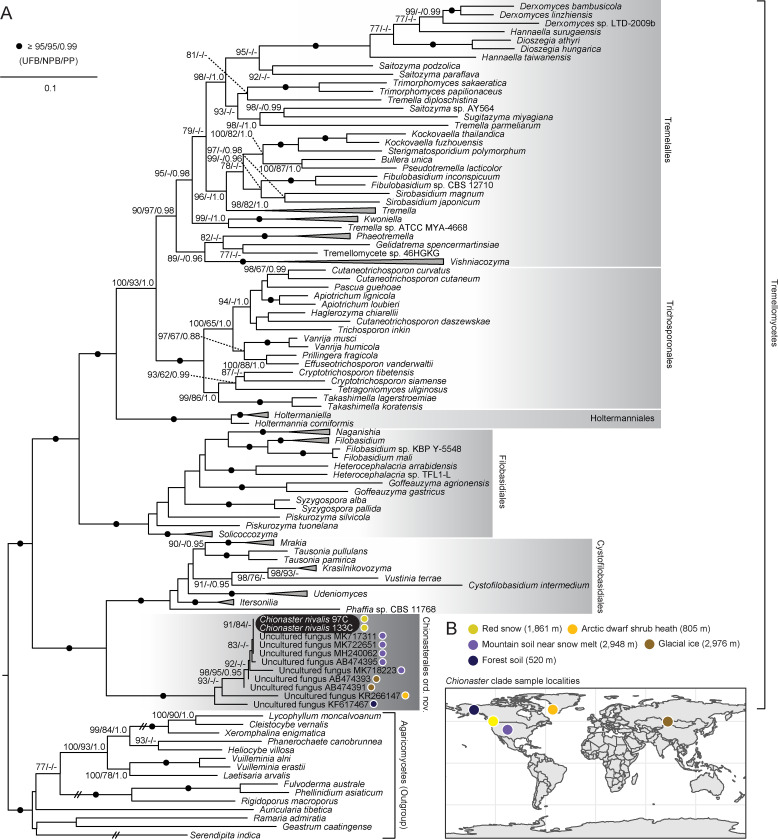
Phylogenetic placement of *Chionaster nivalis*. A maximum-likelihood phylogeny was generated based on a concatenation of the internal transcribed spacer (ITS) and D1-D2 region of the LSU rRNA gene from *C*. *nivalis*, related Tremellomycetes, environmental sequences, and an Agaricomycete outgroup, using the SYM+R6 nucleotide substitution model [43]. All species are represented by both the ITS and the D1-D2 with the exception of certain uncultured environmental fungi and *C*. *nivalis* 97C (see S1 Table). Statistical support is shown at each node and was generated from 1000 ultrafast bootstraps (UFB), 1000 non-parametric bootstraps (NPB), and Bayesian posterior probabilities (PP). Values above 95 UFB, 95 NPB, and 0.99 PP are indicated with black circles whereas values below 75 UFB, 75 NPB, and 0.95 PP are not shown. *Chionaster nivalis* is highlighted in black and the five recognized Tremellomycete orders have been outlined in grey. Sample localities for each sequence branching within the Chionasterales have been denoted with coloured circles. For clarity certain branch lengths have been reduced by 50% as denoted by the hashed lines and clades comprising over three representatives of exclusively the same genera were collapsed and shown as wedges. See S1 Table for a list of taxa and their respective accession numbers. **B.** A map illustrating the locations in which members of the Chionasterales were identified. Sample types and altitudes are denoted. Altitudes were estimated using FreeMapTools.com.

Concatenation of the ITS and D1-D2 regions followed by maximum likelihood and Bayesian phylogenetic analyses indicate that *C*. *nivalis* is a member of the Basidiomycota and in particular, the Agaricomycotina (Fig 2A, S1 Fig). This affiliation was statistically supported even when more distantly related outgroups such as Ustilaginomycetes and Wallemiomycetes were included in the analysis (S1 Fig). Despite previous hypotheses, *C*. *nivalis* is distantly related to the Chytridiomycota indicating that the globules observed within the central cell are likely not zoospores (Figs 1B, 1G and 2A). Likewise, despite morphological similarities to aquatic hyphomycetes (which are primarily ascomycetes), *C*. *nivalis* was not closely related to any previously sampled species, including a recently described basidiomycetous hyphomycete, *Classicula sinensis*, which belongs to the distantly related Pucciniomycotina [38]. The aquatic hyphomycetes have previously been shown to be polyphyletic suggesting that the hyphomycete morphology is convergent [33, 34, 39], and indeed, *C*. *nivalis* represents an additional independent transition to a hyphomycete morphology. The star-like appearance of aquatic hyphomycetes is typically associated with fast moving freshwater habitats where the shape can encourage snaring and substrate retention [40–42]. This could indicate that the structure of *C*. *nivalis* may be important for positioning within the snow layer or may affect interactions with run-off and melt water.

Within the Agaricomycotina, *C*. *nivalis* was placed within the Tremellomycetes with strong statistical support (Fig 2A). However, *C*. *nivalis* did not fall within any of the five previously described Tremellomycete orders (Tremellales, Trichosporonales, Holtermanniales, Filobasidiales, or Cystofilobasidiales) (Fig 2A) [44]. Instead, *C*. *nivalis* formed a distinct, independent, order-level clade with a small group of environmental sequences from uncharacterized species that branched sister to the Cystofilobasidiales with full statistical support (Fig 2A). Likewise, *C*. *nivalis* also had the highest BLAST (Basic Local Alignment Search Tool) similarity to representatives of the Cystofilobasidiales within the non-redundant NCBI (National Centre for Biotechnology Information) nucleotide database. This divergent phylogenetic position fits with the unusual morphology of *C*. *nivalis* and may explain why the species has been challenging to classify in the past. Although the phylogenetic resolution of the ITS and D1-D2 permitted the confident placement of the *Chionaster*-containing clade relative to other Tremellomycete orders, future phylogenomic analyses based on the sequencing of additional loci will be required to corroborate this topology. This is important given the phylogenetic ambiguity that resulted from the inclusion of more distant outgroups, indicating that the topology can be influenced by long branch attraction (S1 Fig). Nonetheless, morphological and phylogenetic distinctiveness relative to other Tremellomycetes warrants the description of both a novel order and family to accommodate *C*. *nivalis*, described here as the Chionasterales ord. nov. (MB838717) and the Chionasteraceae fam. nov. (MB838718). The placement of *C*. *nivalis* within the Tremellomycetes also indicates that it may represent a portion of sequences previously identified in environmental DNA surveys as basidiomycetous yeast, and reclassification of these datasets may permit finer level taxonomic classifications of fungal communities and more comprehensive biogeographical characterizations of *C*. *nivalis*.

In addition to reference taxa, the environmental sequences we identified in the NCBI non-redundant nucleotide database to be closely related to *C*. *nivalis* are also informative, even though the source organisms are not identified. Phylogenetic analysis revealed that a number of environmental sequences generated from fungal ITS and LSU clone libraries were confidently placed within the Chionasterales with full statistical support (26–99.5% alignment coverage for individual loci) (Fig 2A). Sequence identity between environmental sequences and *C*. *nivalis* varied from 89.6%-100% indicating the likely presence of multiple species, genera, and perhaps families within the Chionasterales. Moreover, all of the related environmental sequences were derived from either high altitude or arctic localities including soil and ice in the mountains of Colorado and Siberia, as well as forest soil and plant heath in Alaska and Greenland, respectively (Fig 2B). The absence of related sequences from alternative environments suggests that species within this order are likely psychrophilic, but are not strictly limited to snow habitats, although it is possible that certain life stages may depend on local environmental conditions. The establishment of cultures will be an important next step for experimentally assessing the optimal growth temperature and psychrophilicity of *C*. *nivalis*. Additionally, the distribution of the Chionasterales confirms that it is globally distributed, in accordance with previous microscopic records [9, 21, 24–28].

## Conclusions

*Chionaster nivalis* is a frequently observed and widely distributed constituent of microbial snow communities that has remained phylogenetically and taxonomically enigmatic since its discovery in 1893 [32]. Here, we characterized the ITS and D1-D2 region from *C*. *nivalis*, which will not only serve as molecular barcodes for future studies but permitted its identification as a Tremellomycete, and a representative of a previously undescribed order that is sister to the Cystofilobasidiales. These results not only emphasize the unrecognized diversity that exists within these extreme environments but also highlight the utility of using single-cell isolation approaches for classifying members of complex communities and the growing need to develop culture techniques for extremophiles. Further ultrastructural, life cycle, and ecological observations will be required to properly characterize the Chionasterales, and molecular identification of other frequently observed snow-associated fungi, namely *C*. *bicornis* and *S*. *nivalis*, will be important for understanding the diversity of these organisms. The global range of *C*. *nivalis* also makes it a useful model for understanding biogeography and species distributions, and its phylogenetic position and relation to other snow and ice-associated genera, such as *Naganishia* and *Mrakia*, suggest it could be valuable for understanding cold-adaptation in fungi and other eukaryotes more broadly.

## Taxonomy

**Chionasterales** N. A. T. Irwin, C. S. Twynstra, V. Mathur, P. J. Keeling, **ord. nov.** MycoBank MB838717.

Member of the Tremellomycetes. The diagnosis of the order Chionasterales is based on the genus *Chionaster*. The nomenclature of the order is based on the genus *Chionaster*.

Type family: Chionasteraceae N. A. T. Irwin, C. S. Twynstra, V. Mathur, P. J. Keeling.

**Chionasteraceae** N. A. T. Irwin, C. S. Twynstra, V. Mathur, P. J. Keeling, **fam. nov.** MycoBank MB838718.

Member of the Chionasterales. The diagnosis of the family Chionasteraceae is based on the genus *Chionaster*. The nomenclature of the family is based on the genus *Chionaster*.

Type genus: *Chionaster* Wille, Nytt Magazin for Naturvidenskapene 41: 97–187 (1903); MB22081.

Genus accepted: *Chionaster* Wille, Nytt Magazin for Naturvidenskapene 41: 97–187 (1903).

***Chionaster*** Wille, Nytt Magazin for Naturvidenskapene 41: 97–187 (1903). **emend**. N. A. T. Irwin, C. S. Twynstra, V. Mathur, P. J. Keeling.

The genus is emended to include the species *Chionaster nivalis* based on molecular phylogenetic analysis of the ribosomal DNA operon and is assigned to the family Chionasteraceae, within the order Chionasterales of the Tremellomycetes. The original description of *Chionaster* was based on the presence of three to five radiating arms and a central condensed cell (i.e., an aplanospore) and lacked higher level taxonomic classifications. Phylogenetically the genus is placed in the Chionasterales of the Tremellomycetes, as revealed by phylogenetic analyses (Fig 2). Members of the genus are geographically widespread and have been detected in arctic and high alpine environments (Fig 2).

Type species: *Chionaster nivalis* (Bohlin) Wille, Nytt Magazin for Naturvidenskapene 41: 97–187 (1903); MB560972.

## Materials and methods

### Sample collection and microscopy

Samples of red snow were collected adjacent to Joffery Lake (1,600m) and Wedgemount Lake (1,861m) on the 30th May 2018 and the 28th June 2019, respectively. Debris free red snow was collected in 50 mL conical tubes prior to storage at 4˚C. Microscopic observations on the melted snow were made using either a Leica DM IL inverted microscope or a Zeiss Axioplan 2 microscope using differential interference contrast. The nuclear morphology of *Chionaster nivalis* was observed after incubating the cells for 2 hours in 8 μg/mL DAPI (4′,6-diamidino-2-phenylindole). Fluorescent excitation was achieved using an X-Cite 120LED illuminator and photomicrographs were acquired using a Sony A7RIII mounted using an LMScope Digital SLR Universal Adapter.

### Isolation of *Chionaster nivalis*

Given the complexity of the microbial communities found in red snow, *Chionaster nivalis* needed to be concentrated and isolated from contaminants. To this end, samples of melted snow were initially diluted to 25% (500 μL melted snow: 1.5 mL deionized water) and passed through a 10 μm-PluriSelect cell strainer in order to reduce the abundance of small particles and bacterial cells. The strainer was subsequently washed three times with 1 mL deionized water before the retentate was collected with 200 μL deionized water. Individual *C*. *nivalis* cells were then isolated from the strained samples using glass capillary micropipettes, washed twice in deionized water, and combined into pools of 97 or 133 cells. To further account for potential contamination issues, negative controls were collected by mimicking the cell isolation protocol but without isolating *C*. *nivalis* cells.

### DNA extraction and sequencing

To ensure cell fracture and lysis during DNA extraction, pooled cells and control samples were initially disrupted through freeze thaw cycles in liquid nitrogen. Microcentrifuge tubes containing the pooled isolates were placed in liquid nitrogen for 30 seconds before being removed and allowed to thaw for three minutes at room temperature. After five freeze-thaw cycles, DNA extraction was performed using a DNeasy Power Biofilm Kit (Qiagen) which includes a ten-minute bead-beating step using an OMNI Bead Ruptor, which promoted further disruption. After DNA extraction, the internal transcribed spacer (ITS) region of the ribosomal RNA operon and the D1-D2 region of the large subunit ribosomal rRNA gene, both useful phylogenetic markers for fungal species identification, were amplified by polymerase chain reaction (PCR) using ITS1-F (5’–CTTGGTCATTTAGAGGAAGTA A– 3’) and NLB3-R (5’–GGATTCTCACCCTCTATGA– 3’) primers for the ITS and NL1 (3’–GCATATCAATAAGCGGAGGAAAAG– 5’) and NL4 (5’–GGTCCGTGTTTCAAGACGG– 3’) for the D1-D2, in combination with Phusion High Fidelity PCR Master Mix (New England Biolabs) [36, 37, 45]. PCR was conducted over 35 cycles according to the manufacturer’s instructions with an annealing temperature of 55˚C and an extension time of 60 seconds. Whereas the D1-D2 amplicon was sequenced directly, PCR products obtained for the ITS were cloned using a StrataClone Blunt PCR cloning kit (Agilent Technologies) and inserts were analyzed by colony PCR using M13F (5’-TGTAAAACGACGGCCAGT-3’) and M13R (5’-CAGGAAACAGCTATGACC-3’) primers in combination with EconoTaq Plus Green PCR Master Mix (Lucigen). Amplicons were sequenced on both strands by Sanger sequencing conducted by GeneWiz. The resulting sequences were imported into Geneious v7.1.3 (Biomatters) and assembled into contigs following the removal of low-quality bases from both the 5’ and 3’ ends. Consensus sequences for the ITS were obtained from a minimum of three clones.

### Phylogenetic analysis

Newly derived *Chionaster nivalis* sequences were firstly compared to the NCBI (National Centre for Biotechnology Information) non-redundant nucleotide database using BLASTn [46] before being integrated into ITS and D1-D2 datasets containing a comprehensive set of reference Tremellomycete taxa previously collated by Li et al. (2020) [47] (S1 Table). The datasets were supplemented with Agaricomycetes, Wallemiomycetes, and Ustilaginomycetes as outgroups (S1 Table).

To conduct phylogenetic analyses, multiple sequence alignments were first generated for both the ITS and D1-D2 datasets in MAFFT v.7.222 using the L-INS-I algorithm [48] before being trimmed using trimAl v1.2 with a gap-threshold of 30% and visualized in AliView v1.26 [49, 50]. The trimmed ITS and D1-D2 alignments were subsequently concatenated and maximum-likelihood phylogenies were determined using IQ-Tree v2.0.3 [51]. When more distant outgroups were included, 20% of the sites with the fastest substitution rates, calculated in IQ-Tree, were removed to reduce the effects of long branch attraction. Statistical support was generated from 1,000 ultrafast and non-parametric bootstraps [51, 52]. Substitutions models were selected based on Bayesian Information Criteria in ModelFinder [53]. Bayesian analyses were conducted in MrBayes v3.2.6 over two runs, each consisting of four chains (three heated and one cold), for ten million generations using the default burn-in of 25% and the GTR+I+G4 substitution model as selected by ModelTest [54, 55]. Phylogenies were visualized and annotated using FigTree v1.4.2 [56].

## Supporting information

S1 Fig Phylogenetic analysis of *Chionaster nivalis* and related Agaricomycetes, Wallemiomycetes, and Ustilaginomycetes. A maximum-likelihood phylogeny generated using a concatenation of the internal transcribed spacer (ITS) and D1-D2 region of the LSU rRNA gene using the SYM+I+G4 nucleotide substitution model following the removal of the 20% fastest evolving sites, as inferred in IQ-Tree, to limit long branch attraction. The included taxa are similar to those included in Fig 2 with the addition of Wallemiomycetes, Ustilaginomycetes, and additional Agaricomycetes which represent more distant outgroups for the Tremellomycetes. All species are represented by both the ITS and the D1-D2 with the exception of certain uncultured environmental fungi and *C*. *nivalis* 97C (see S1 Table). Statistical support is shown at each node and was generated from 1000 ultrafast bootstraps (UFB), 1000 non-parametric bootstraps (NPB), and Bayesian posterior probabilities (PP). Values above 95 UFB, 95 NPB, and 0.99 PP are indicated with black circles whereas values below 75 UFB, 75 NPB, and 0.95 PP are not shown. *Chionaster nivalis* is highlighted in black and the five recognized Tremellomycete orders have been outlined in grey. For clarity, clades comprising over three representatives of exclusively the same genus or representing the Tremellales, Trichosporonales, and Holtermanniales were collapsed and are shown as wedges. See S1 Table and Fig 2 for a list of taxa and their respective accession numbers.
(EPS)

S2 Fig (TIF)

S1 Table Accession numbers for sequences included in the phylogenetic analyses. (XLSX)
