# Supplementary figures and images for: The molecular phylogeny of Chionaster nivalis reveals a novel order of psychrophilic and globally distributed Tremellomycetes (Fungi, Basidiomycota)
Source: PLoS One. 2021 Mar 24;16(3):e0247594. doi: 10.1371/journal.pone.0247594 (PMC7990227; doi:10.1371/journal.pone.0247594)

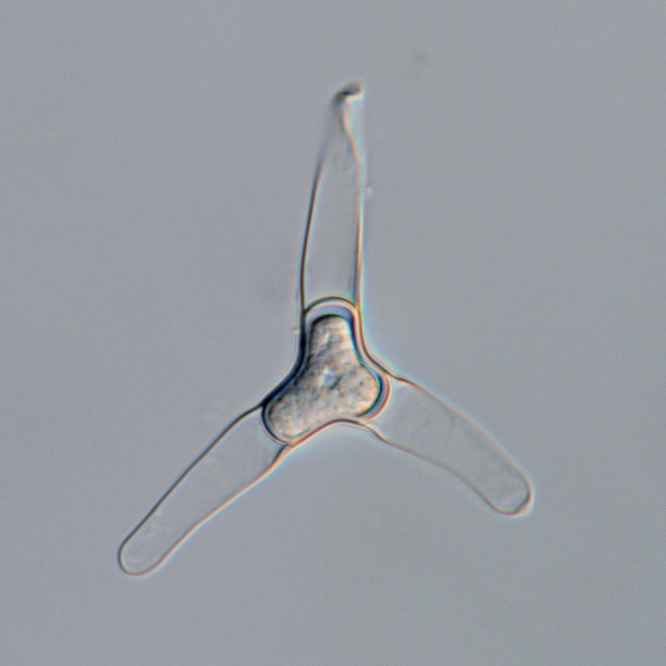

Supplement: S2 Fig — (TIF) [file pone.0247594.s002.tif]
